# Preservation Obscures Pelagic Deep-Sea Fish Diversity: Doubling the Number of Sole-Bearing Opisthoproctids and Resurrection of the Genus *Monacoa* (Opisthoproctidae, Argentiniformes)

**DOI:** 10.1371/journal.pone.0159762

**Published:** 2016-08-10

**Authors:** Jan Yde Poulsen, Tetsuya Sado, Christoph Hahn, Ingvar Byrkjedal, Masatoshi Moku, Masaki Miya

**Affiliations:** 1 Fish Section, Australian Museum, Sydney NSW, Australia; 2 Department of Fish and Shellfish, Greenland Institute of Natural Resources, Nuuk, Greenland; 3 Natural History Museum and Institute, 955–2 Aoba-cho, Chuo-ku, Chiba, Japan; 4 School for Biological, Biomedical and Environmental Science, University of Hull, Hull, United Kingdom; 5 Natural History Collections, Bergen Museum, University of Bergen, Bergen, Norway; 6 Atmosphere and Ocean Research Institute, The University of Tokyo, 5-1-5 Kashiwano-Ha, Kashiwa, Chiba, Japan; Southwest University, CHINA

## Abstract

The family Opisthoproctidae (barreleyes) constitutes one of the most peculiar looking and unknown deep-sea fish groups in terms of taxonomy and specialized adaptations. All the species in the family are united by the possession of tubular eyes, with one distinct lineage exhibiting also drastic shortening of the body. Two new species of the mesopelagic opisthoproctid mirrorbelly genus *Monacoa* are described based on pigmentation patterns of the “sole”—a unique vertebrate structure used in the reflection and control of bioluminescence in most short-bodied forms. Different pigmentation patterns of the soles, previously noted as intraspecific variations based on preserved specimens, are here shown species-specific and likely used for communication in addition to counter-illumination of down-welling sunlight. The genus *Monacoa* is resurrected from *Opisthoproctus* based on extensive morphological synaphomorphies pertaining to the anal fin and snout. Doubling the species diversity within sole-bearing opisthoproctids, including recognition of two genera, is unambiguously supported by mitogenomic DNA sequence data. Regular fixation with formalin and alcohol preservation is shown problematic concerning the retention of species-specific pigmentation patterns. Examination or photos of fresh material before formalin fixation is shown paramount for correct species recognition of sole-bearing opisthoproctids—a relatively unknown issue concerning species diversity in the deep-sea pelagic realm.

## Introduction

Opisthoproctid fishes or Barreleyes constitute one of the most peculiar and unknown fish groups in the deep-sea pelagic realm, with only 19 morphologically disparate species currently described in the family Opisthoproctidae [1]. The opisthoproctids is one of four families in the protacanthopterygian order Argentiniformes, including also Bathylagidae, Microstomatidae and Argentinidae [2]. Marine smelts in the family Argentinidae is a likely candidate for an extant opisthoproctid sister clade although phylogenetic relationships within Argentiniformes are poorly resolved at present [3–5]. All the 19 opisthoproctid species are united by tubular dorsally directed eyes, although the fishes are showing very different morphologies and body plans. However, due to the relative rareness and fragility of most species, the answers to numerous questions regarding life history, taxonomy, deep-sea pelagic adaptations, and general biology in the group remain as of yet elusive. Although the only vertebrates known that uses a mirror instead of a lens to focus an image are included within the Opisthoproctidae [6–7], anatomical studies related to the exceptional visionary adaptations of most opisthoproctid species are still missing [8]. There is also an evolutionary shortening of the body in this family, that have terminated in several species exhibiting unusual deep-bodied morphs, not usually witnessed in deep-sea fishes.

**Fig 1:**
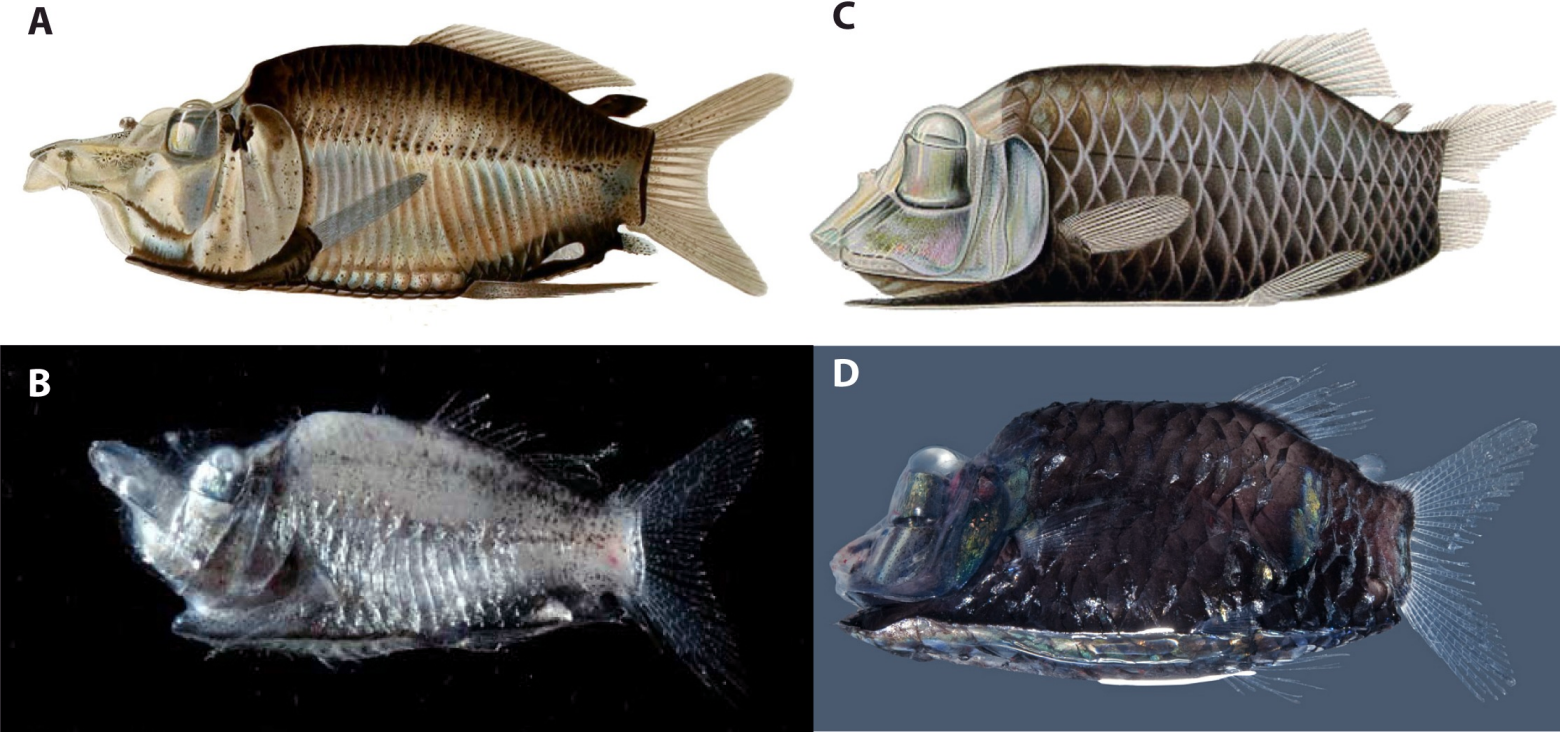
**Illustrations and photographs of freshly caught sole-bearing opisthoproctids (A–D).** A, *Monacoa grimaldii*, after Zugmayer (1911). B, *M*. *grimaldii*, ZMUB 18926, freshly caught, photo by D. Shale. C, *Opisthoproctus soleatus*, after Brauer (1906). D, *O*. *soleatus*, photo by S. Johnson. Note the large snout and developed anal fin in *M*. *grimaldii* separating the two genera.

**Fig 2:**
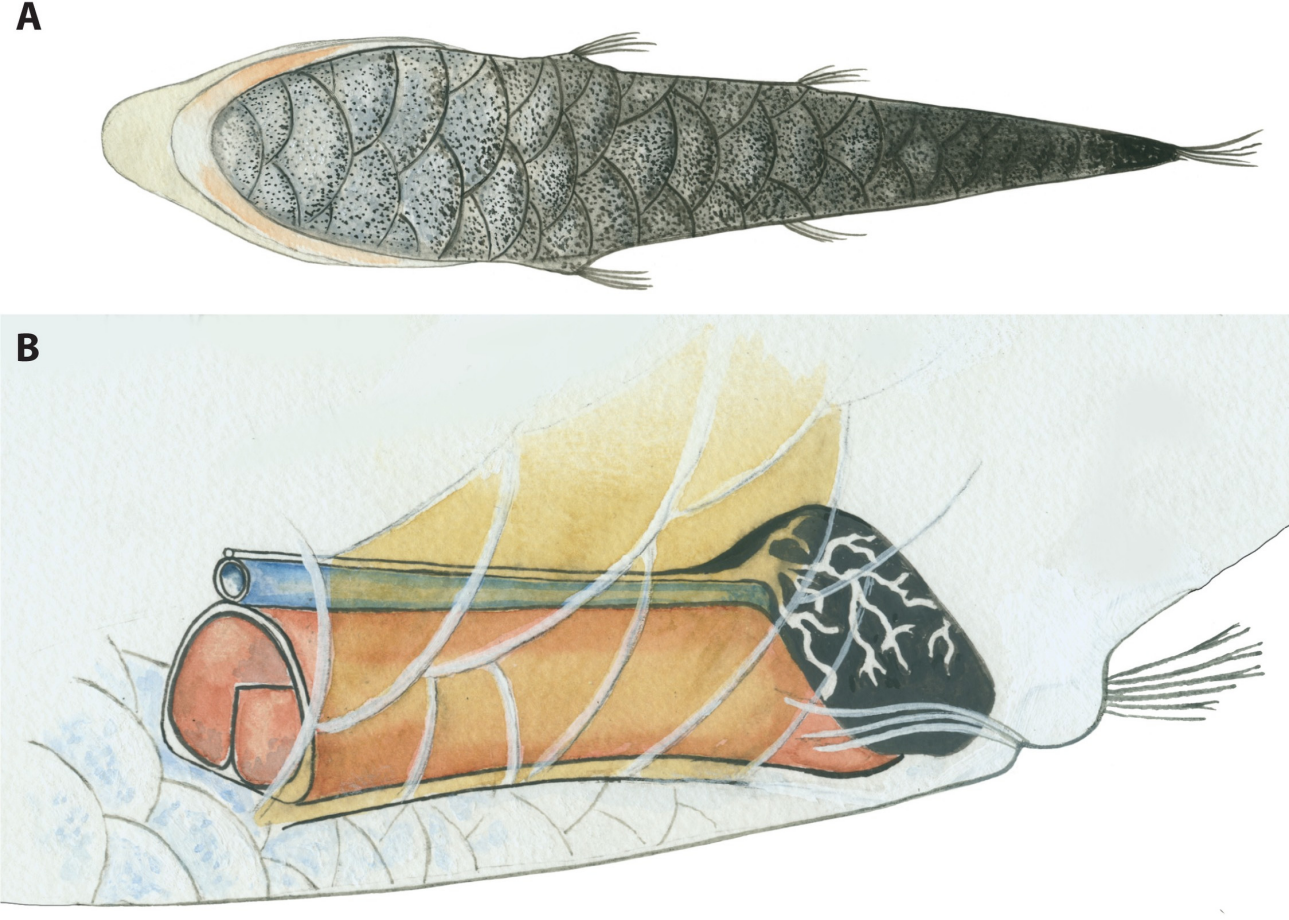
**Illustrations of the “sole” and hyaloid body (reflector) found in species of the genus *Monacoa* (A–B).** A, The sole—a large flattened structure covered with large pigmented scales. B, Light organ and hyaloid body (reflector), controlling bioluminescence. Bioluminescence is produced in the large black light organ, a modification of the distal intestinal tract, situated above the anal opening. Modified for *Monacoa* after Bertelsen & Munk [12].

The specialized tube-eyes in the genus *Opisthoproctus* currently comprise two species (Fig 1): the barreleye *Opisthoproctus soleatus* described by Vaillant [9] and the mirrorbelly *Opisthoproctus grimaldii* described by Zugmayer [10]. They are united by a unique vertebrate synaphomorphy, the ventral “sole”. The sole constitutes a large flat area as wide as the maximum body width and as long as the entire trunk region (Fig 2A). The ventral margin of fishes is usually tapered towards a narrow ridge along the entire ventral part; however, in these fishes, this ventral space is enlarged and has evolved into a large reflector organ that controls light emission. Light is produced in the “rectal bulb”, a modification of the distal part of the intestinal tract close to the anus, and channeled through a small opening into an internal reflector-lumen called the hyaloid body (Fig 2B). The entire external surface of the sole is covered with large thin scales showing gradually increasing pigmentation toward the distal parts, thereby functioning as a light-screen when the reflector is contracted (no light emission) or expanded (light passes through the thin transparent parts of the scales) [11–12]. Bioluminescence is produced in the rectal bulb via bacteria [13] and although the photogenic structure and location in other ophistoproctids differ somewhat between genera, such as the “anal bulb” of *Rhynchohyalus natalensis*, separating it in origin and position from the rectal bulb, different sections of the rectal mucous membranes have evolved into bioluminescent producing structures in opisthoproctids [14]. External ventrally positioned luminous tissues are also present in other opisthoproctids, such as *Dolichopteryx*, and even in other argentiniforms such as microstomatids of the genus *Nansenia* [15]. However, these structures, including bioluminescent tissue in argentiniforms, are poorly known, and apparently only present in certain life-stages in some species [16].

Although united by the sole, the two *Opisthoproctus* species show striking differences concerning the snout and in the configuration of the anal fin (Fig 1). These differences convinced Chapman [17] of generic status and he erected *O*. *grimaldii* to *Grimaldia grimaldii*, although renamed to *Monacoa grimaldii* by Whitley [18] as the former name was linked to a previously described genus of amphipods. The only subsequent change to the classification of sole-bearing opisthoproctids occurred when Cohen [19] removed the genus name *Monacoa*, largely because only two sole-bearing opisthoproctids were known and therefore rendering generic status trivial. Considering the substantial morphological differences between the two previously recognized species, in combination with the two additional species presented in the current study, and including unambiguous molecular support, the generic status of *Monacoa* is resurrected and *Opisthoproctus* is from here on only used in reference to *O*. *soleatus*.

The most closely related taxon to the genera *Monacoa* and *Opisthoproctus* is the Barreleye *Macropinna microstoma* described by Chapman [20]. The latter species famed opisthoproctid fishes from in situ ROV observations off California, showing for the first time a transparent integument shield covering the inside situated dorsally directed eyes with green lenses. The tubular eyes are being protected by a fluid-filled chamber of unknown substance, plausibly screening the eyes from the stinging cells of siphonophores [21]. *Macropinna microstoma* presents the only other “short bodied” opisthoproctid in the family, although it lacks the unique ventral sole of *Opisthoproctus* and *Monacoa*. All opisthoproctid species apparently possess the transparent shield and the amazing footage of the Barreleye brought to life extreme morphological adaptations, mesmerizing ichthyologists and layman viewers alike, and proving that we know only little about the pelagic deep-sea and its peculiar although highly adapted inhabitants. The two new species described here are testaments to the latter statement, with sole pigmentation patterns shown to be species specific, although previously identified as mere intraspecific variation [22]. All photophore-possessing deep-sea pelagic fish groups are known to communicate by bioluminescent or phosphorescent flash signaling [23], although artifacts related to standard formalin fixation and alcohol preservation concerning species identification has not received much attention in deep-sea organisms. The fixative formalin is an aqueous solution of formaldehyde and hereafter noted only as formalin. Light production and species recognition in meso- and bathypelagic animals are aided by numerous morphological constructions regulating light emission, and evidence is presented in this study that show the sole-bearing opisthoproctids to be no exception. We here show preservation an important caveat in the identification of sole-bearing opisthoproctids, doubling the species diversity in this group of peculiar fishes, owing to examination and comparisons of fresh and formalin fixed material.

## Material and Methods

### Morphology

Considering the results presented in this study, that species delimiting characters of *Monacoa* spp. are sole patterns of pigmentation, reexamination of the two syntypes was unnecessary for this study. No designated holotype is present [24]. Sole patterns of pigmentation are often obscured by long time preservation, and the comprehensive morphometric and sole-pattern notes on both syntypes of *M*. *grimaldii* by Zugmayer [10], are unambiguous concerning this matter. Measurements were done with a digital caliper to the nearest 0.1 mm and digital X-ray images were done at the Australian Museum (AMS) in Sydney, Australia, and at the National Science Museum Tokyo (NSMT) in Tsukuba, Japan. All type material was caught with *R/V Hakuho-Maru* during the research cruise KH-13-7 in 2013–14, Atmosphere and Ocean Research Institute (AORI), University of Tokyo, Japan, with a 10-feet IKMT with mesh size of 5 mm. All sampling was conducted in international waters. No permits were required. All type material was preserved in 99.5% ethanol onboard and kept in sub-zero temperatures, a good practice concerning preservation of form and pigmentation. Species delimitations of *Monacoa* spp. are largely drawn from this fresh material in this study, as pigmentation patterns of the soles are intact, and clearly obscured in comparative material by long-time ethanol preservation following formalin fixation. Comparative Atlantic material, fixated in 70% ethanol onboard, for morphological and molecular examination were provided from the Zoological Museum University of Bergen (ZMUB), Norway, caught during the Atlantic ridge MARECO-Eco cruise in 2004 [25]. A photo by D. Shale (MARECO), taken immediately after capture onboard, is included for comparisons of sole pigmentation patterns, thereby having pigmentation patterns of non-formalin fixed material for all three species of *Monacoa*. Comparative material from the west Pacific Ocean, that have been long-time preserved and without tissue available for DNA comparisons, were provided from the AMS. We decided not to include formalin-fixed material from the eastern Pacific and Indian Oceans, in order not to confuse results further as preservation obscures pigmentation patterns considerably. I.e., including additional material from these regions proved indifferent to this study.

Nomenclatural Acts: The electronic edition of this article conforms to the requirements of the amended International Code of Zoological Nomenclature, and hence the new names contained herein are available under that Code from the electronic edition of this article. This published work and the nomenclatural acts it contains have been registered in ZooBank, the online registration system for the ICZN. The ZooBank LSIDs (Life Science Identifiers) can be resolved and the associated information viewed through any standard web browser by appending the LSID to the prefix “http://zoobank.org/”. The LSID for this publication is: urn:lsid:zoobank.org:pub: 713EDAB6-040A-4067-A842-DE592A82F29A. The electronic edition of this work was published in a journal with an ISSN, and has been archived and is available from the following digital repositories: PubMed Central and LOCKSS.

#### Type material

(Fig 3)

**Fig 3:**
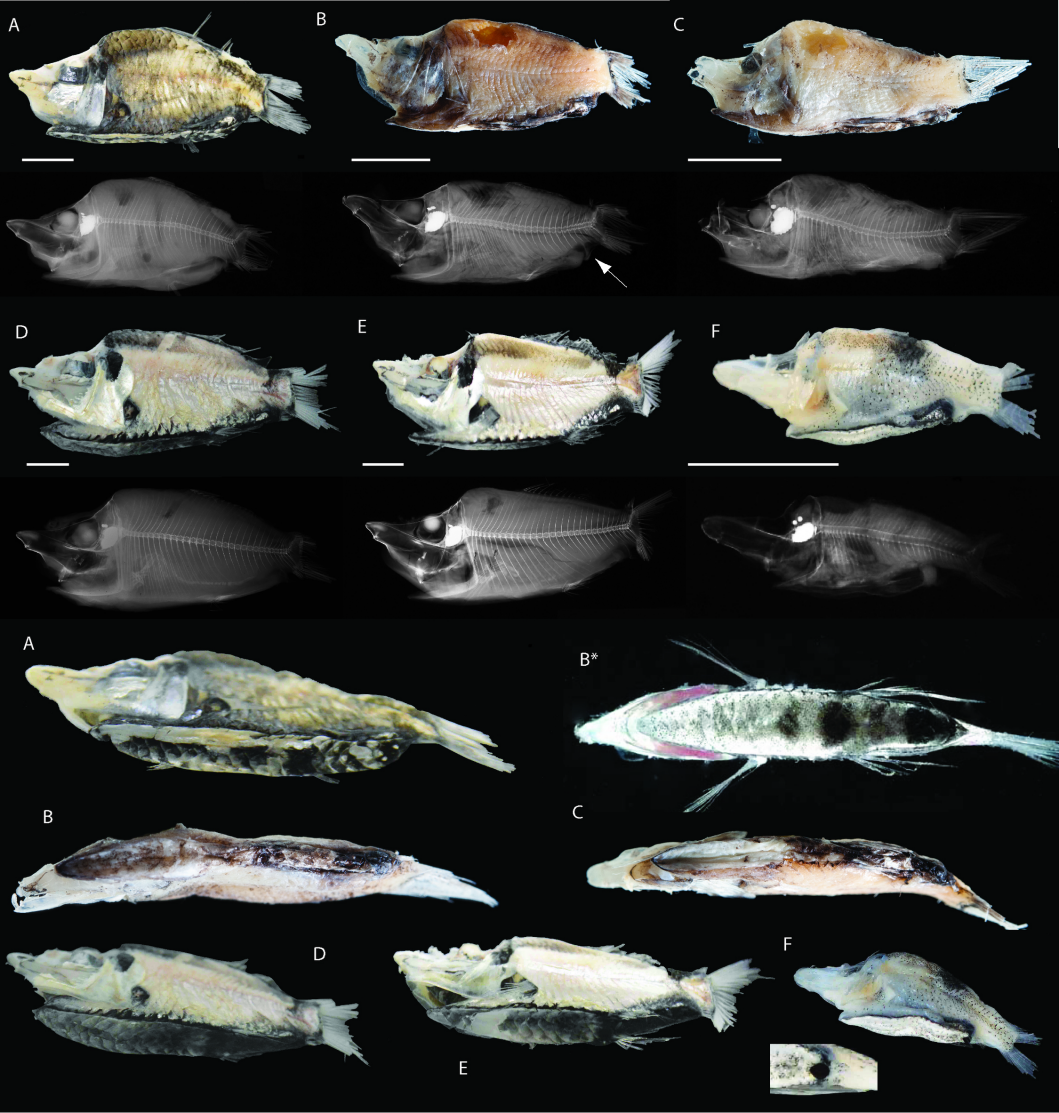
Photographs, X-rays and sole pigmentation patterns of type and comparative material included for both morphological and molecular comparisons in this study. **Ethanol fixation used for all specimens–type material 99.5% and comparative material 70%. Scale bars equal 10 mm (A–F).** A, *Monacoa niger*, new species, holotype, CBM-ZF 14683. Note the strong pigmentation along the entire sole (anterior part partly damaged). B, *Monacoa grimaldii*, ZMUB 18926, (B* newly caught with specimen shown in Fig 1B). C, *M*. *grimaldii*, ZMUB 18977 (anterior part partly damaged). D, *Monacoa griseus*, new species, holotype, CBM-ZF 14677. E, *M*. *griseus*, new species, paratype, CBM-ZF 14681 (anterior part partly damaged). F, *M*. *griseus*, new species, paratype, CBM-ZF 14757 (juvenile). Insert shows protruding rectal organ being particular distinct in juveniles compared to adults.

*Monacoa niger* sp. nov. Holotype (unique). Natural History Museum and Institute, Chiba (CBM-ZF 14683), 48.0 mm SL unsexed specimen. Holotype caught December 24–25 2013, 22:43–00:40 local time, north-north-east off American Samoa (05°05'S, 170°03'W) at depth 0–520 m (station 2).

*Monacoa griseus* sp. nov. Holotype. Natural History Museum and Institute, Chiba (CBM-ZF 14681), 63.9 mm SL unsexed specimen. Holotype caught January 13 2014, 17:20–19:15 local time, east of New Zealand (40°06'S, 169°58'W) at depth 0–521 m (station 9).

Paratype. Natural History Museum and Institute, Chiba (CBM-ZF 14677), 64.5 mm SL unsexed specimen. Paratype caught January 13 2014, 17:20–19:15 local time, east of New Zealand (40°06'S, 169°58'W) at depth 0–521 m (station 9).

Paratype. Natural History Museum and Institute, Chiba (CBM-ZF 14757), 17.8 mm SL unsexed specimen. Paratype caught January 13 2014, 17:20–19:15 local time, east of New Zealand (40°06'S, 169°58'W) at depth 0–521 m (station 9).

#### Comparative material including molecular sequences

(Fig 3)

*Monacoa grimaldii*. Zoological Museum University of Bergen, Bergen (ZMUB 18926), 28.6 mm SL unsexed specimen. Caught June 28, 2004, at 42°47'N, 29°32'W off the mid-Atlantic Ridge in the North Atlantic at fishing depth 0–795 m.

Zoological Museum University of Bergen, Bergen (ZMUB 18977), 34.0 mm SL unsexed specimen. Caught June 30, 2004, at 41°42'N, 29°60'W off the mid-Atlantic Ridge in the North Atlantic at fishing depth 205–684 m.

#### Comparative material without molecular sequences

(Fig 4)

**Fig 4:**
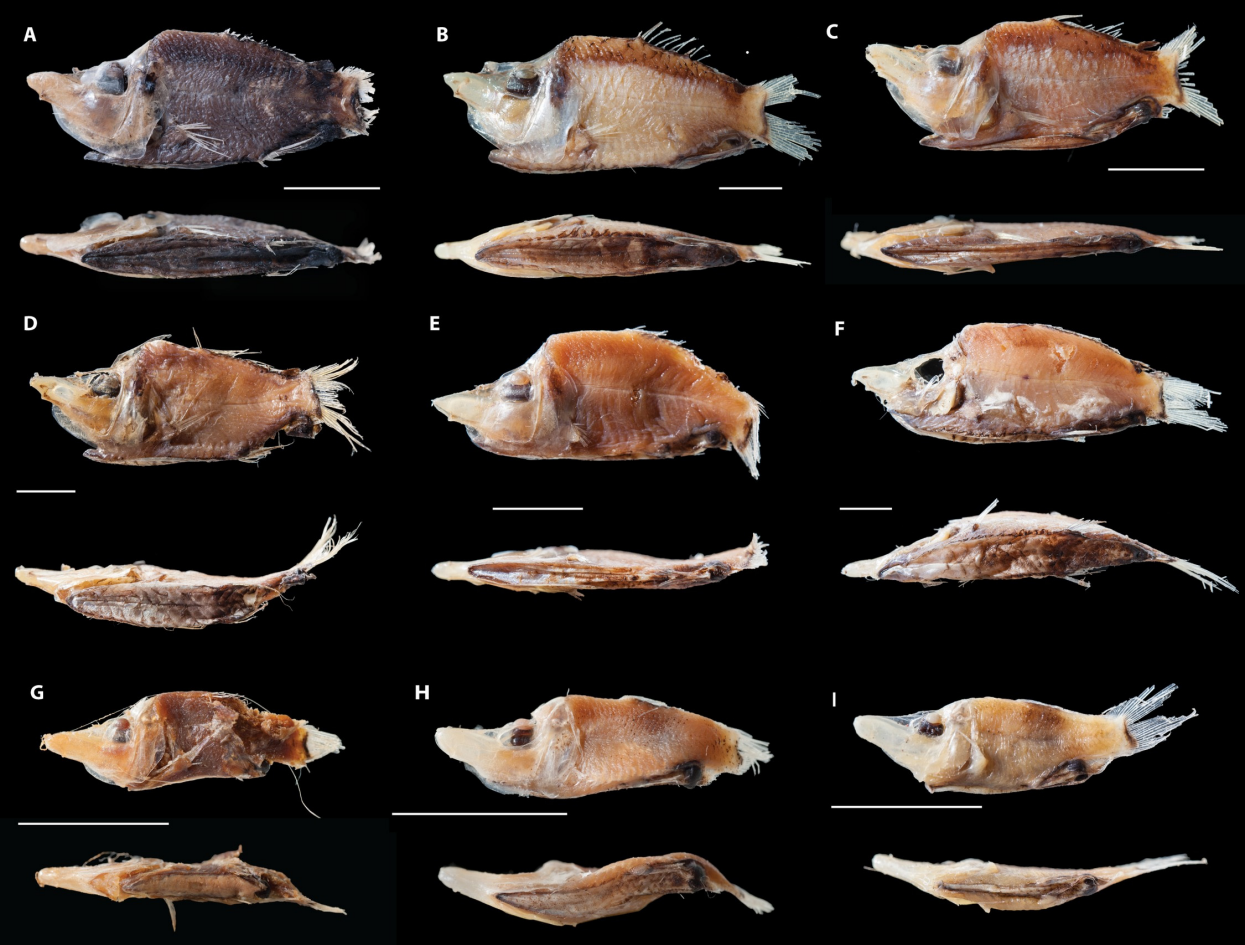
**Adult formalin fixed and alcohol preserved specimens of *Monacoa* spp. (A–F), and juvenile formalin fixed and alcohol preserved specimens of *Monacoa* spp. (G–I).** The following species designations (“cf”) are tentative. A, *Monacoa* cf. *niger*, AMS I.25919-001. B, *Monacoa* cf. *griseus*, AMS I.21744-001. C, *M*. cf. *griseus*, AMS I.16494-030. D, *M*. cf. *griseus*, AMS I.21366-001. E, *M*. cf. *griseus*, AMS I.16492-025. F, *M*. cf. *griseus*, AMS I.24031-002. G, *M*. cf. *niger*, juvenile, AMS I.20942-001. H, *M*. cf. *griseus*, juvenile, AMS I.19751-022. I, *M*. cf. *griseus*, juvenile, AMS I.21748-001.

*Monacoa* cf. *niger*. AMS I.20942-001, 17.1 mm SL (East of Cape York, Queensland, Australia); AMS I.25919-001, 33.1 mm SL (East of Crowdy Head, New South Wales, Australia).

*Monacoa* cf. *griseus*. AMS I.21748-001, 15.9 mm SL (East of Botany Bay, New South Wales, Australia); AMS I.19751-022, 17.3 mm SL (33°20'S, 152°17'E); AMS I.21748-001, 17.7 mm SL (East of Botany Bay, New South Wales, Australia); AMS I.20064-058, 25.7 mm SL (East of Sydney, New South Wales, Australia); AMS I.16492-025, 33.3 mm SL (East of Sydney, New South Wales, Australia); AMS I.16494-030, 33.6 mm SL (East of Sydney, New South Wales, Australia); AMS I.21744-001, 47.4 mm SL (East of Wilsons Promontory, Victoria, Australia); AMS I.21366-001, 49.8 mm SL (East of Newcastle, New South Wales, Australia); AMS I.24031-002, 56.0 mm SL (East of Newcastle, New South Wales, Australia).

### Molecular sequences and phylogenetic analyses

Mitogenomes (complete mitochondrial genomes) were determined for all three species of *Monacoa* in addition to *Macropinna microstoma*, *Dolichopteryx minuscula* and *Bathylychnops exilis*. Mitogenomic data of other argentiniform and protacanthopterygian fishes used in this study, were previously determined [5, 15, 26] (Table 1). Depending on tissue quality, mitogenomes were determined either with a long PCR amplification technique [27] and subsequently nested short PCR and sanger-sequenced, or next generation sequencing using the MiSeq Sequencing platform (Illumina) at Natural History Museum and Institute, Chiba. For the latter MiSeq sequencings, all libraries were prepared from the long PCR products, using Nextera XT DNA Library Preparation Kit following manufacture´s protocol. Argentiniform fishes show a canonical mitochondrial gene order [28] and universal fish primers for both the long and short nested PCRs can be employed if material are not degraded by bad preservation [29–30]. Gene annotation was performed using tRNA-scan-SE ver. 1.21 [31] and by alignment to closely related species previously determined for their mitogenomes. Trimming of the MiSeq reads was performed with the MIRA ver. 4.0.2 assembler (http://sourceforge.net/p/mira-assembler/wiki/Home/) and paired end reads were merged using FLASH [32]. Assembly of mitogenomes was performed using MITObim ver. 1.8 with default settings [33] and Sequencher ver. 5.0.1 (Gene codes). A Jupyter notebook illustrating the Illumina data processing and assembly is available in S1 File. All DNA sequences were deposited in the DDBJ/EMBL/GenBank databases and labeled according to GenSeq for type material [34]. The 13 protein coding gene sequences contained in the mitogenome were aligned by eye whereas the 22 tRNA genes, 12S and 16S rRNA genes were aligned using ProAlign [35] including only sites with posterior probabilities of 90% or higher. The resultant dataset, including a total of 16,202 base pairs, was sectioned into five partitions (-q option) corresponding to the 1^st^, 2^nd^ and 3^rd^ codon positions of the protein coding genes, the 4^th^ partition consisting of the 22 concatenated tRNAs genes and the 5^th^ consisting of the two concatenated rRNA gene sequences. The dataset was analyzed using maximum likelihood (ML) method, using the sequential version of the software RAxML ver. 8.1.17 [36]. A single run searching for the best scoring ML-tree, including 1000 bootstrap replicates, was specified using the -f a and–# options, respectively. Model of sequence evolution was the GTR+G+I as found by ModelGenerator ver. 0.85 [37].

**Table 1:** Specimen information for taxa included in this study with mitogenomic DNA sequence data.

Species	Museum	NCBI [study]
**Argentiniformes**		
**Argentinidae**		
*Argentina silus*	---	AP012952 [15]
*Glossanodon semifasciatus*	---	AP004105 [5]
**Bathylagidae**		
*Bathylagus ochotensis*	---	AP004101 [5]
**Opisthoproctidae**		
*Bathylychnops exilis*	NSMT P73392	AP012953 (this study)
*Dolichopteryx minuscula*	NSMT P68890	AP012954 (this study)
*Macropinna microstoma*	SIO 09–334	AP017327 (this study)
*Monacoa grimaldii*	ZMUB 18977	AP017325 (this study)
*Monacoa grimaldii*	ZMUB 18926	AP017323 (this study)
*Monacoa griseus*	CBM-ZF 14677	AP017328 (this study)
*Monacoa griseus*	CBM-ZF 14681	AP017326 (this study)
*Monacoa griseus*	CBM-ZF 14757	AP017324 (this study)
*Monacoa niger*	CBM-ZF 14683	AP017322 (this study)
*Opisthoproctus soleatus*	---	AP004110 [5]
**Microstomatidae**		
*Nansenia ardesiaca*	---	AP004106 [5]
*Nansenia boreacrassicauda*	---	AP012955 [15]
**Esociformes (Esocidae)**		
*Esox niger*	---	AP013046 [26]
**Galaxiiformes (Galaxiidae)**		
*Galaxias maculatus*	---	AP004104 [5]
**Salmoniformes (Salmonidae)**		
*Parahucho perryi*	---	AP013048 [26]

## Results

### Diagnosis of the genus *Monacoa* [10]

Adults: Body short and laterally compressed; ventral surface flattened into a reflecting sole, covered with multiple thin overlapping variously pigmented scales; body scales large, deciduous and cycloid; eyes tubular and directed dorsally with large lenses visible when viewed from above; eyes cradled in suborbitals and postorbitals; nasal capsules present dorsally on head; nostrils paired, raised by papillae supported by paired nasal bones lateral to the capsules; snout long, approximately 20% SL; skull of roof transparent and gelatinous, brain visible; no photophore on eye tube; no suborbital light organs; bony interorbital area reduced to a thin sliver; suborbitals large, laterally shielding eyes and cheeks; lateral line straight, running along the entire body, with 27–35 scales, not reaching base of caudal; opercle large and deep; preopercle large and L-shaped, ventral arm reaching anterior to the vertical of eye; anal fin present albeit small, obliquely or vertically positioned on posterior outer margin of sole, easy discernible and clearly separated from both caudal and pelvic bases; branchiostegal rays 2; premaxillae absent; maxillae a scale-like bone, easily lost; dentary scoop-like, lacking teeth; cleithrum supporting the anterior part of the sole; urohyal projecting downwards, supporting anterior part of sole; large bone of unknown origin (possibly a modified radial or inter-haemal bone) supporting anal fin and the posterior part of sole (see X-rays in Fig 3); dorsal fin base long, approximately 25% SL; large, fully pigmented adipose fin present, slightly smaller than anal fin; pectoral fin rays long, reaching bases of pelvics; pectoral bases angled, situated laterally on body on peduncles, slightly closer to sole than to lateral line; pelvic fins long reaching behind anus, situated laterally, not within the sole; sole length approximately 80% SL; anus at posterior end of sole; rectal organ present on distal part of intestine, immediately underneath anus; skin strongly pigmented around anus and anal base; caudal fin broad-based, large and deeply forked, fin rays always broken; body color pigmentation (scale pockets) variable from light to dark hues, preservation important; dorsal part of the epaxial area separated, showing darker scale pockets compared to more ventrally, more prevalent in some species than others (Figs 1, 3 and 4).

**Fig 5:**
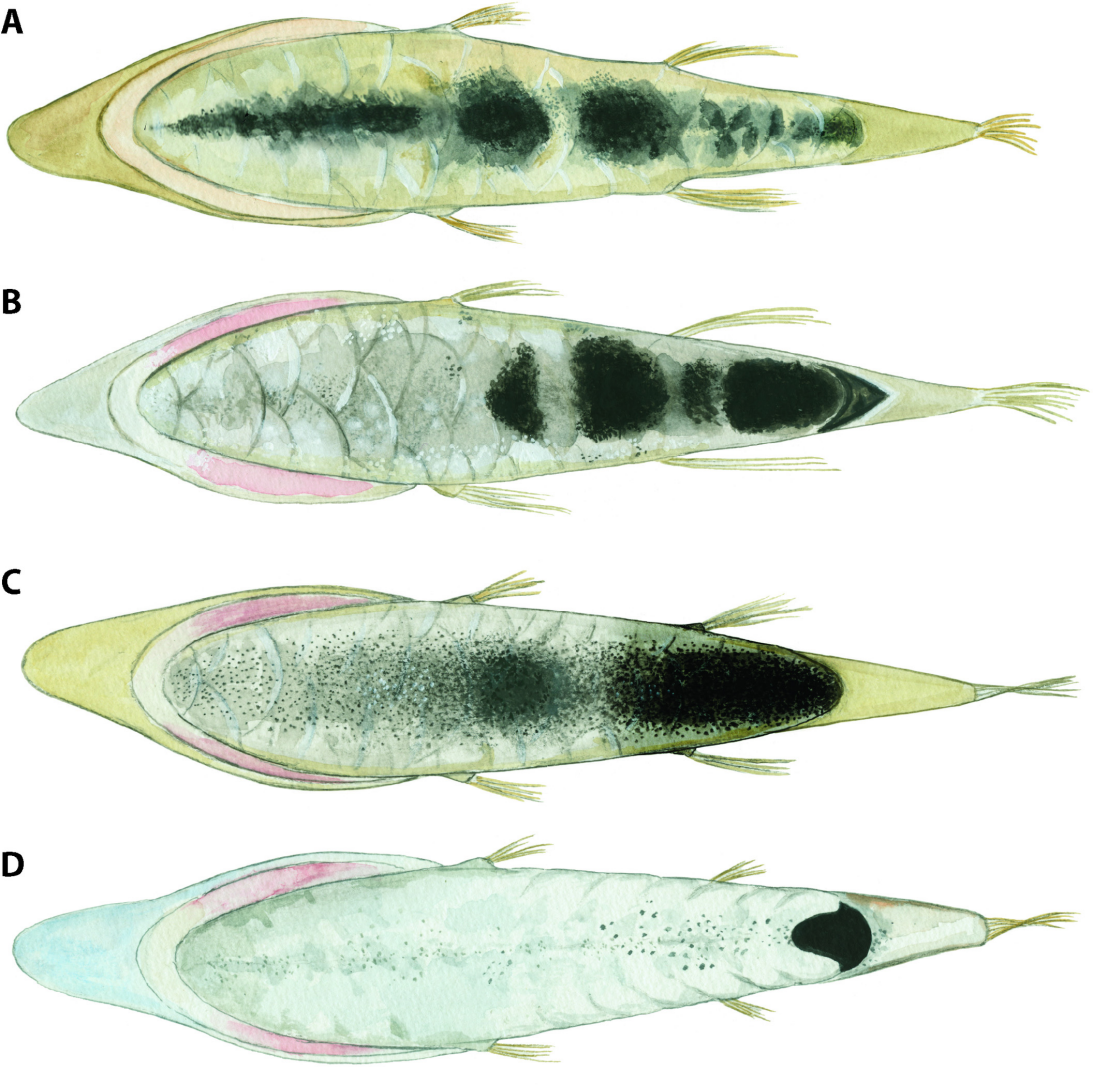
**Illustrations of sole pigmentation patterns found in the genus *Monacoa* (A–D).** A, *M*. *niger*. B, *M*. *grimaldii*. C, *M*. *griseus*. D, *M*. *griseus* (juvenile). Juveniles of all *Monacoa* species are likely appearing similar to *M*. *griseus* illustrated here.

Juveniles (only characters different from the adults are listed): characters based on CBM-ZF 14757 (17.8 mm SL), AMS I.21748-001 (15.9 mm SL), AMS I.20942-001 (17.1 mm SL), AMS I.19751-022 (17.3 mm SL), AMS I.21748-001 (17.7 mm SL), a 14.0 mm SL specimen discussed by Cohen [38], two “Michael Sars” specimens (15.2 and 19.0 mm SL) and one 22.6 mm SL specimen in a separate note by Cohen [19]; snout comically large and distinct in width, length and girth; length of sole smaller as compared to adults, increasing in % SL with growth, length ranging from 50–70% SL; sole uniform, largely iridescent in fresh/non-formalin preserved material, with scattered tiny pigmentations without distinct patterns (Fig 5); large black irregular blotch present between dorsal base and lateral line; anal fin relative horizontal positioned, developing into a oblique or vertical position in adults; anus bulb/rectal organ protruding, likely more distinct in juveniles compared to adults.

Etymology: The genus name *Monacoa* is re-erected in this study and was constructed by Whitley [18] without giving any etymological reason for the chosen name. However, it is likely referring to the state of Monaco in which the research expedition that sampled the two syntypes originated.

Suggested vernacular name: Long-nosed mirrorbellies. Japanese: Hikari-deme-nigisu.

Size: Species of *Monacoa* are deep-bodied and relatively small fishes with a maximum SL of 64.5 mm for *M*. *griseus* reported at present (this study). The total lengths are considerably longer although caudal fin rays always damaged.

*Monacoa niger* sp. nov.: This species shows black sole pigmentation along the entire sole and possibly have a darker or more blackish body color compared to congeners (scale pockets are darker). The black streak on the sole possibly consists of distinct patches posteriorly (Fig 3A and Fig 5A; Table 2). The holotype is slightly damaged on the sole and variation of the sole pigmentation pattern in this species must be assessed with future fresh material.

**Table 2:** Data on type material of *Monacoa* spp. Four different sole pigmentation patterns have currently been observed within *Monacoa* (A–D) and are explained in the results section.

Species	*Monacoa niger*	*Monacoa griseus*	*Monacoa griseus*	*Monaco griseus*	*Monacoa grimaldii*	*Monacoa grimaldii*
Specimen	CBM-ZF 14683	CBM-ZF 14677	CBM-ZF 14681	CBM-ZF 14757	MOM 0091–1163	MOM 0091–1163
Status	Holotype	Holotype	Paratype	Paratype	Syntype	Syntype
Position	W Pacific	SW Pacific	SW Pacific	SW Pacific	N Atlantic	N Atlantic
TL^a^	57.5	72.1	71.2	20.8	---	---
SL	48.0	64.5	63.9	17.8	48.0	44.0
% SL						
HL	45.4	43.7	42.3	43.3	43.8	43.2
Predorsal	57.9	60.2	53.2	56.2	64.6	65.9
Preanal	91.9	93.0	90.9	77.5	---	---
Prepelvic	69.2	66.8	67.3	62.9	72.9	72.7
Snout length	19.2	18.6	18.2	22.5	20.8	20.5
Horizontal eye diameter	10.2	11.3	10.5	---	12.5	12.5
Dorsal fin base length	28.5	25.6	24.4	25.3	---	---
Anal fin base length	4.0	3.5	5.0	6.7	---	---
Width at eye	14.2	14.7	15.2	11.2	10.4	10.2
Depth at eye	33.5	34.4	31.5	22.5	---	---
Depth pectoral fin	45.8	45.0	39.3	36.0	39.6	43.2
Width pectoral fin	16.3	15.8	15.8	15.2	14.6	14.8
Caudal peduncle depth	16.7	14.9	13.8	16.3	15.6	15.9
Sole length (incl. anus)	76.6	82.2	78.1	54.5	---	---
Pre-caudal vertebrae	15	15	15	---	---	---
Caudal vertebrae	19	19	20	---	---	---
Dorsal fin rays	14	14	14	14	14	14
Anal fin rays	8	9	9	9	8	8
Pectoral fin rays	11	11	11	11	14	14
Pelvic fin rays	10	12	12	11	14	14
Caudal fin rays	38	36	34	37	37	37
Lateral line scales	26	26	29	---	27	27
Longest scale row on sole	26	26	27	---	---	---
Gill rakers	19	20	25	---	---	---
Pseudobranch filaments	8	13	11	---	---	---
Sole pattern	A	C	C	D	B	B
Dorsolateral spot	No	No	No	Yes	No	No

^a^ Caudal fin rays fragile and damaged in all specimens examined.

CBM-ZF 14683 (holotype). GenBank/GenSeq: AP017322/genseq-1 mitogenome.

ZooBank LSID: urn:lsid:zoobank.org:act:C7619C68-C720-43D8-9400-AFEF7E9BE840.

*Monacoa griseus* sp. nov.: This species shows a greyish anterior part of the sole, abruptly changing just in front of the pelvic fins, to a dense pigmented posterior part. The two adult specimens of this species (Fig 3D–3E; Fig 5C; Table 2) show in addition a relatively weak irregular large blotch just in front of the change in pigmentation, centered approximately below the anterior part of the dorsal fin in the vertical plane. Scale pockets are possibly relatively light.

CBM-ZF 14681 (holotype). GenBank/GenSeq: AP017326/genseq-1 mitogenome.

CBM-ZF 14677 (paratype). GenBank/GenSeq: AP017328/genseq-2 mitogenome

CBM-ZF 14757 (paratype). GenBank/GenSeq: AP017324/genseq-2 mitogenome

Note: CBM-ZF 14757 is a juvenile, confirmed as *M*. *griseus* from identical molecular DNA sequences. Juveniles discussed below.

ZooBank LSID: urn:lsid:zoobank.org:act:1E9DDCC2-0AC2-41E6-93B5-DB9398D34058.

#### Comparative material including molecular sequences

(Fig 3; Table 3)

**Table 3:** Data on comparative material of *Monacoa* spp. Only ZMUB specimens included with molecular data. All specimens, included with “cf”, are tentative due to formalin fixation possibly changing pigmentation patterns. Sole patterns A–D correspond to Fig 5.

Species	*Monacoa* cf. *niger*	*Monacoa* cf. *niger*	*Monacoa* cf. *griseus*	*Monacoa* cf. *griseus*	*Monacoa* cf. *griseus*	*Monacoa* cf. *griseus*	*Monacoa* cf. *griseus*	*Monacoa* cf. *griseus*	*Monacoa* cf. *griseus*	*Monacoa* cf. *griseus*	*Monacoa* cf. *griseus*	*Monacoa grimaldii*	*Monacoa grimaldii*
Specimen	AMS I.20942-001	AMS I.25919-001	AMS I.21748-001	AMS I.19751-022	AMS I.21748-001	AMS I.20064-058	AMS I.16492-025	AMS I.16494-030	AMS I.21744-001	AMS I.21366-001	AMS I.24031-002	ZMUB P18977	ZMUB P18926
Position	SW Pacific	SW Pacific	SW Pacific	SW Pacific	SW Pacific	SW Pacific	SW Pacific	SW Pacific	SW Pacific	SW Pacific	SW Pacific	N Atlantic	N Atlantic
TL^c^	20.0	35.3	20.9	20.5	22.1	32.4	38.1	38.2	56.9	62.3	69.8	40.3	35.4
SL	17.1	33.1	15.9	17.3	17.7	25.7	33.3	33.6	47.4	49.8	56.0	34.0	28.6
% SL													
HL	46.8	41.4	42.1	46.2	42.9	40.5	44.4	42.6	46.2	46.6	40.5	40.6	38.8
Predorsal	61.4	57.1	58.5	64.7	61.6	56.4	59.2	57.4	56.1	57.0	57.7	60.0	60.1
Preanal	78.9	93.3	84.9	90.8	86.4	92.6	88.3	92.6	94.9	95.4	94.5	92.1	90.6
Prepelvic	70.2	66.8	62.3	65.9	68.9	62.3	66.7	63.1	69.4	67.1	66.3	63.2	59.4
Snout length	23.4	19.6	22.6	24.3	19.8	21.0	18.3	18.5	20.7	20.7	20.0	20.6	22.4
Horizontal eye diameter	12.9	12.4	13.2	11.0	14.7	10.1	13.8	14.6	11.4	11.2	10.4	11.8	11.2
Dorsal fin base length	24.6	25.4	24.5	24.3	26.0	26.5	25.8	26.5	26.2	28.5	27.3	25	23.8
Anal fin base length	6.4	2.7	6.9	4.0	6.2	3.1	3.9	4.2	3.6	3.2	2.9	5.6	4.2
Width at eye	14.6	14.2	14.5	16.2	15.3	12.5	13.8	15.5	14.3	14.7	10.9	14.4	10.8
Depth at eye	28.7	31.1	27.7	26.0	33.3	24.5	28.2	33.6	34.4	32.9	27.9	34.1	31.1
Depth pectoral fin	35.1	41.4	38.4	36.4	39.5	36.6	40.2	40.8	42.6	43.4	37.1	40.3	40.6
Width pectoral fin	15.8	14.8	16.4	17.3	18.1	14.4	14.1	17.0	17.7	17.7	16.1	13.2	13.6
Caudal peduncle depth	18.7	15.7	17.6	16.7	14.7	16.7	16.8	19.3	16.7	15.7	14.8	16.2	19.2
Sole length (incl. anus)	56.1	76.1	59.7	68.8	63.3	---	77.5	75.3	78.9	78.3	81.6	74.1	81.5
Pre-caudal vertebrae	---	15	---	---	---	15	15	14	15	15	15	14	14
Caudal vertebrae	---	19	---	---	---	19	19	20	20	20	19	17	17
Dorsal fin rays	14	13	---	14	---	14	14	14	14	14	14	15	14
Anal fin rays	---	8	---	8	---	---	8	8	8	8	8	8	8
Pectoral fin rays	---	12	---	---	---	11	12	12	11	12	12	12	12
Pelvic fin rays	---	11	---	11	---	12	11	10	10	10	10	13	12
Caudal fin rays	32	37	35	34	34	33	34	36	35	35	37	32	33
Lateral line scales	---	27	---	---	---	25	---	25	27	26	---	26	28
Longest scale row on sole	---	26	---	---	26	---	---	25	29	25	26	---	26
Gill rakers	---	18	---	---	---	21	23	20	26	24	24	23	21
Sole pattern	D	A	D	D	D	---	---	---	C	C	C	---	B
Dorsolateral spot	Yes	No	Yes	Yes	Yes	No	No	No	No	No	No	No	No

*Monacoa grimaldii*. ZMUB 18926. GenBank: mitogenome AP017323

ZMUB 18977. GenBank: mitogenome AP017325

#### Pigmented sole-patterns in *Monacoa*

Four sole pigmentation patterns (A–D) have been identified in the genus *Monacoa* from this study and we have designated them as follows; A) a black streak of pigmentation, likely patchy at the posterior end, running along the entire sole, possibly covering most of the sole, although variation not presently known (*M*. *niger*—Fig 5A); B) four-spot pattern, including two distinct larger patches in between two weaker smaller patches, all located at the posterior part of the sole (*M*. *grimaldii*—Fig 5B); C) greyish uniform sole pattern in the anterior part, with a stronger tint in the anterior-posterior direction, with the posterior part abruptly turning much darker, with a poorly defined weaker patch present immediately in front of the abrupt change (*M*. *griseus*—Fig 5C); D) uniform sole with minute scattered pigmentations in no distinct patterns, appearing iridescent in fresh material (currently observed in all juveniles across species—Fig 5D). Preservation is an important caveat concerning patterns of sole pigmentation and is discussed below. The sole pigmentation patterns of all species and the juvenile are illustrated in Fig 5.

Etymology: *Monacoa niger* sp. nov. The species derivative is originating from Latin “niger” (black) referring to the black streak of pigmentation present on the sole. Suggested vernacular name: black mirrorbelly. Japanese: kuro-hikari-deme-nigisu.

Etymology: *Monacoa griseus* sp. nov. The species derivative is originating from Latin “griseus” (grey) referring to the uniform greyish anterior part of the sole lacking distinct patterns of pigmentation. Suggested vernacular name: grey mirrorbelly. Japanese: haiiro-hikari-deme-nigisu.

**Key to “sole” opisthoproctids (adults):**

1a Snout slightly pointed, relatively rounded, not protruding into tube (10–15% SL); anal fin retrorse, often absent; sole length approx. 90% SL; dorsal fin base approx. 20% SL................................................ ***Opisthoproctus soleatus***
1b Snout prominent, protruding into distinct tube (18–25% SL); anal fin present, easily discernible, situated on posterior outer margin of sole; sole length approx. 80% SL; dorsal fin base approx. 25% SL (genus *Monacoa*)................ 2
2a Streak of black pigmentation present along entire sole, patchy at posterior end (Fig 5A);.................................. ***Monacoa niger* sp. nov**
2b Anterior part of sole without distinct pigmentation, posterior part darkly pigmented or showing distinct pigmented blotches........................ 3
3a Posterior part of sole with four irregular black blotches of pigmentation, the second and fourth much bigger than first and third in anterior-posterior direction (Fig 5B)......................... ***Monacoa grimaldii***
3b Posterior part of sole darkly/uniformly pigmented, with abrupt change in pigmentation in front of pelvic fins, including weaker pigment blotch in front of abrupt change in pigmentation (Fig 5C).. ***Monacoa griseus* sp. nov.**

### Mitogenomic phylogeny

**Fig 6:**
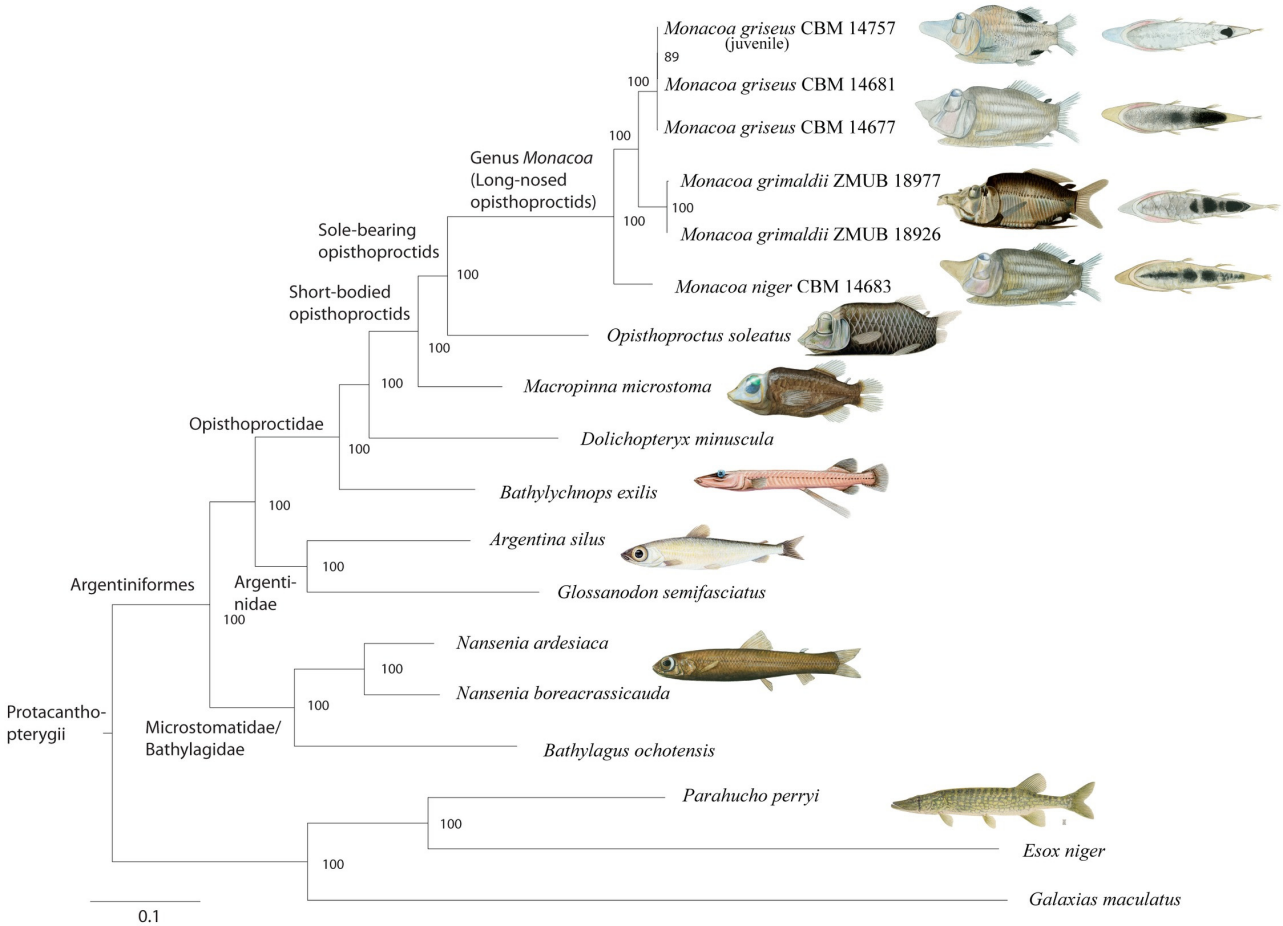
Mitogenomic phylogeny of Argentiniformes and sole-bearing opisthoproctids using other protacanthopterygians as outgroups. Fish illustrations included for all short-bodied opisthoproctids and other representative protacanthopterygians, including all sole pigmentation patterns currently recognized in the genus *Monacoa*. Note the relatively large molecular distance separating the two genera *Opisthoproctus* and *Monacoa*, supporting generic status based on morphology.

The protacanthopterygian mitogenomic ML-phylogenetic tree shows the family Opisthoproctidae sister family to the Argentinidae. Short-bodied opisthoproctids are found monophyletic with sole-bearing opisthoproctids representing the sister clade to *Macropinna microstoma*. The three species of *Monacoa* are found relatively distinct from other ophistoproctids, including *Opisthoproctus soleatus*, with interspecific edge lengths ranging between 4–6% within *Monacoa*. The juvenile specimen included shows almost 100% DNA sequence similarity to adults of *M*. *griseus*, with only 12 (0.07%) and 14 (0.08%) base pairs differences comparing the whole mitogenomes. The Atlantic species *M*. *grimaldii* and the Pacific species *M*. *griseus* are supported as sister taxa, both constituting a sister clade to the Pacific *M*. *niger*. All nodes are supported by bootstrap values of 100 except a node denoting two specimens of *M*. *griseus* showing almost complete DNA sequence identity (Fig 6).

### Distributions

**Fig 7:**
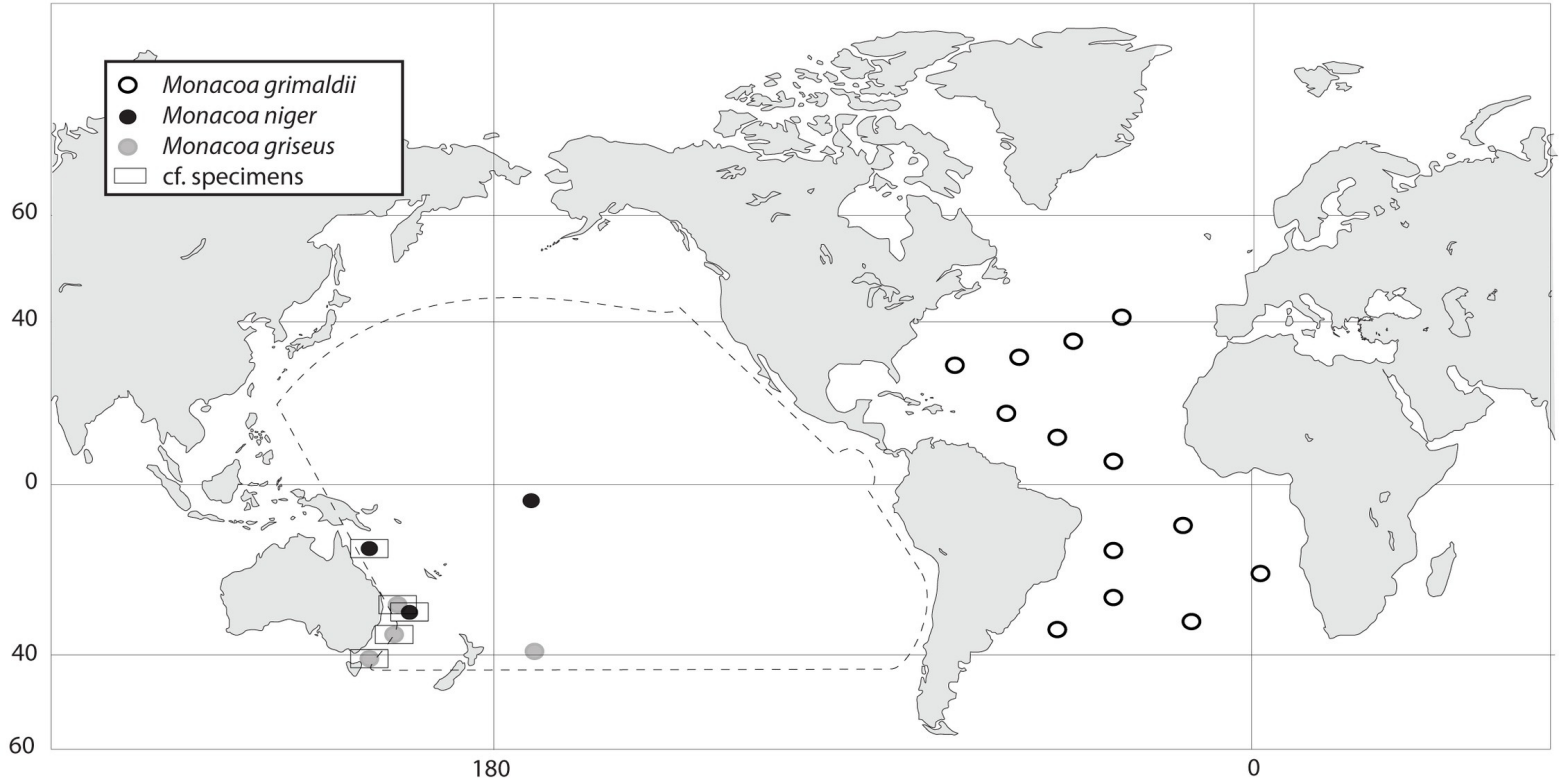
Occurrence of *Monacoa* spp. including tentative distributions of *Monacoa niger* and *Monacoa griseus* (truncated area). Squared records are tentatively identified from formalin fixed material (Fig 4). Reprinted from http://freevectormaps.com [39] under a CC BY license, with permission from T. Grajeda (http://freevectormaps.com).

Indications are that *M*. *grimaldii* is the only species present in the Atlantic Ocean between approximately 45°N and 45°S, *M*. *griseus* is apparently the common *Monacoa* species in the southwestern Pacific Ocean and *M*. *niger* is a tropical Pacific species, although the latter is only confidently known from the holotype at present. The two AMS specimens identified as *M*. cf. *niger* in this study are both from the southwestern Pacific (Figs 4 and 7). However, formalin fixed/alcohol preserved material are currently ambiguous concerning proper species identification (see below) and future molecular data and examination of fresh specimens with intact sole patterns should determine distributions more adequately.

The depth ranges reported in past literature include catch data of *Monacoa* in bathypelagic depths below 2000 m. However, no reliable data are present from closing nets and we question the occurrence of these fishes in bathypelagic depths. We note that all material used in this study were caught in mesopelagic depths above 800 m with the exact catch depths unknown.

## Discussion

### Two new species, resurrection of *Monacoa* and preservation

It is now clear from this study that the patterns of pigmentation on the sole of long-nosed opisthoproctids are species-specific (Figs 3–6). Melanophore patterns are therefore likely to function in species recognition, in addition to counter-illumination in these fishes. It is interesting to note that ventrally running “blackish or golden tissue” is present in most opisthoproctid species, usually present between the pelvic fins and the anus. However, little information is present concerning communication and species-specific patterns in this family, due to rareness of specimens often being damaged by the fishing gear. The largely vertical direction of bioluminescence produced by ventrally positioned organs, differentiated by melanophore patterns, show the tubular eyes of all opisthoproctid species adapted to such a vertical field of vision. This is contrasting most other bioluminescent groups in which the species-specific photophore patterns are positioned laterally also showing laterally directed eyes. Dorsally directed tubular eyes have, however, evolved several times and also found for example in the marine hatchetfish genus *Argyropelecus* [8, 40–41]. How communication between individuals is actually happening, considering a dorsal field-of-vision and ventral bioluminescence, remains obscure, although likely happening during repositioning of the whole body.

The possibility of recognizing different species based on variation of the sole pigmentation patterns was mentioned by Cohen [19]. He considered it an adult feature, as two specimens measuring 15.2 and 19 mm SL did not show any sole pigmentation patterns, contrary to all other adult *M*. *grimaldii* material examined [38]. However, the comparisons made by Cohen included only material of *M*. *grimaldii* from the Atlantic Ocean that was compared to *O*. *soleatus*. Similar comparisons including Pacific material, were noted by Bekker [42], although only the four-spot pattern (Fig 5B) found in *M*. *grimaldii* was noted. Since only two species have been recognized until this study, i.e. *M*. *grimaldii* and *O*. *soleatus*, notes on pigmentation patterns have therefore mostly involved the Atlantic distributed species *M*. *grimaldii*. All observations by the latter studies for *M*. *grimaldii* are in agreement with Zugmayer [10] for the two syntypes and the two Atlantic specimens off the mid-Atlantic ridge included in this study (Fig 3). Little Pacific material has therefore been included in previous comparative works on *Monacoa*, although variation in sole pigmentation was noted as “*brown-black*, *blotched or dusky*” by Paxton & Cohen [43] for opisthoproctids in general. Formalin fixed and subsequent alcohol preserved specimens off eastern Australia (Table 3) all show discoloration present, suggesting formalin fixation to obscure or even alter the melanophore sole patterns in *M*. *niger* and *M*. *griseus* (Figs 3 and 4). The alteration of *M*. *grimaldii* sole patterns in preserved material is evident as well, although the ethanol fixation of ZMUB material used in this study is evidently preserving the pigmentation patterns more accurately compared to formalin fixation. However, several formalin fixed specimens of *M*. *griseus* have somewhat retained the greyish anterior and blackish posterior patterns so clearly observed in fresh material (Figs 3 and 4). Therefore, formalin fixation is not necessarily completely damaging the sole pigmentation patterns, although fresh material stored in alcohol in sub-zero temperatures seems to best retain the sole melanophores within a shorter time frame. Standard preservation of short-bodied opisthoproctid specimens has likely obscured the actual species diversity in sole-bearing ophistoproctids for nearly 100 years. The extent of cryptic species diversity in meso- and bathypelagic fish groups caused by preservation is speculative at this point. However, it could involve a considerable number of undescribed species in various pelagic habitats. Routine metabarcoding approaches, on bulk or eDNA samples, should increase our knowledge concerning this unknown diversity in the near future [44].

Allometric growth changes are present in *Monacoa* witnessed in for example the body depth to SL (Tables 1–3). This character was noted by Bekker [42] to separate the two genera of sole-bearing opisthoproctids. However, the inclusion of data on several juveniles in this study shows this character ambiguous.

The light and dark musculatures of *M*. *grimaldii* specimens, preserved in ethanol only, are apparently not taxonomic informative, as this feature is also found in formalin fixed and ethanol preserved specimens of *M*. *griseus*. However, the holotype of *M*. *niger* shows a very different body coloration (scale pockets) freshly caught compared to congeners and probably distinguishes this species on this feature (Fig 3). One formalin fixed specimen is showing a very dark body color (AMS I.25919-001) and one juvenile is also appearing much darker than the other preserved juvenile specimens (AMS I.20942-001). We have tentatively identified them as *M*. *niger* although the body coloration of the different species is also an issue that requires future verification from fresh material and subsequent fixation (Figs 3 and 4). Variation in body color was noted by Bekker [42] although only as variable between *M*. *grimaldii* and *O*. *soleatus*.

Fresh material is therefore needed in order to establish pigmentation and distribution patterns of *Monacoa* spp. more satisfactorily. However, indications are that *M*. *grimaldii* is found in the Atlantic Ocean and *M*. *griseus* and *M*. *niger* are found in the Pacific Ocean. This is supported from pigmentation patterns listed for the type material of *M*. *grimaldii* by Zugmayer (Table 3) that unambiguously show both specimens to have the four-spot pattern (Fig 5B). The two specimens of *M*. *grimaldii* have only 31 verterbrae as opposed to 34 or more in *M*. *niger* and *M*. *griseus*. However, the latter is based on few specimens (Tables 2 and 3). Chapman [17] noted that the differences observed between *M*. *grimaldii* and *O*. *soleatus* warrant generic status and we confirm this notion with two new species of *Monacoa* and mitogenomic data (Figs 3 and 6).

In order to avoid confusion utilizing comparative material degraded by preservation without molecular tissue available, we have not included eastern Pacific and Indian Ocean material for the purpose of this study. Inclusion of additional preserved material would only confuse the species separation at this point (Fig 4). We note that Bekker [42] only listed *O*. *soleatus* from the Indian Ocean, monotypic and considered circumglobal at this point. Considering the mesopelagic habitat of *Monacoa* spp., we expect *M*. *griseus* and *M*. *niger* to be present across the Pacific Ocean.

### Molecular comparisons and phylogenetics

Mitogenomic sequences of all sole-bearing opisthoproctids show unambiguous species separation, and the variation within *Monacoa* spp. throughout the mitochondrial genome is mostly involving third codon positions in the protein coding genes, polymorphisms throughout the control region and in the highly variable regions of the rRNAs genes. Similar patterns in the location of polymorphisms were found between two sister-taxa of deep-sea smooth-heads of the genus *Leptoderma* (Alepocephalidae), from the Atlantic and Pacific Oceans [30]. Mitogenomics is a valuable tool in the delimitation of taxonomic entities from recent speciation events, including also phylogenetic considerations related to biogeography (Figs 6 and 7). Speciation within *Monacoa* appears relatively recent. No fossil horizons of opisthoproctids are known. Comparisons of the *Monacoa* spp. mitogenomes show that any region can be employed for species identification, as for example a short approximately 170 base pairs region of the 12S rRNA gene recently constructed for metabarcoding studies of marine fishes [44]. There is little doubt that short-bodied opisthoproctids comprise a monophyletic group (Fig 6), a fact corroborated also by e.g. the presence of only one type of visual pigment and photoreceptor in these fishes [8]. However, a surprisingly large molecular distance between *Opisthoproctus* and *Monacoa* is observed, as compared to the only other short-bodied opisthoproctid *Macropinna microstoma*, consolidating the generic statuses of both genera. The Pacific *M*. *griseus* and the Atlantic *M*. *grimaldii* are found sister taxa and constitutes a clade that is sister taxa to *M*. *niger*. This is noteworthy and indicates complicated speciation patterns associated with sole pigmentation patterns and geographical distributions, owing to *M*. *niger* and *M*. *grimaldii* tentatively having more elaborate patches of pigmentation as opposed to *M*. *griseus*. However, whether pigmentation patterns are somehow indicative of direction of evolution in sole-bearing opisthoproctids remains obscure, with speciation in the open oceans a complicated issue for several fish groups [45].

### Juvenile versus adult life stages

Striking ontogenetic changes from juvenile to the adult life stage are becoming apparent when viewing the juvenile specimen and adults of *M*. *griseus* (Fig 3D–3F). A large pigmented irregular spot below the dorsal fin above the lateral line is present in the juvenile, identical to a juvenile specimen likely to be *M*. *grimaldii* from the West Atlantic reported by Cohen [38]. The spot could function as a “fake ocellus” deterring predators, appearing as a giant eye when flashed shortly. This is witnessed in a number of independent deep-sea fishes possessing this feature, such as for example some ophidiiforms [46]. However, the spot is irregularly shaped, clearly not round, or even enclosed by a black rim usually found in fake ocelli. The function of the dark patch in juvenile *Monacoa* spp. is therefore unknown at present. Schmidt [47] merely noted it as a remnant of the larval pigmentation based on one juvenile specimen only. However, considering the presence of this feature in all juveniles present in the length range 14.0–22.6 mm SL, this blotch is conspicuous and likely not merely a juvenile remnant of larval pigmentation. Considering the presence of this spot in *M*. *griseus* and *M*. *grimaldii*, the spot is likely present in all *Monacoa* species and possibly in juvenile *Opisthoproctus* as well. The spot-dimensions of the juvenile paratype (CBM-ZF 14757) and three juvenile specimens examined also for sole pigmentation patterns (AMS I.21748-001, I.20942-001 and I.19751-022), all show the width of the juvenile spot a little longer than the height (5/4 approximately). Preservation using formalin fixation is not an issue with the irregular juvenile blotch.

The juvenile snout of *M*. *griseus* is comically and proportionately enormous compared to the adult equivalent concerning width, length and girth (Fig 3D–3F). Large snout function is also speculative at this stage. However, opisthoproctids are likely pelagic throughout most life stages, although adults might be more or less demersal [16]. Large surface area for detecting water movement and organism-emitted substances seems likely at this point.

The juvenile specimen of *M*. *griseus* presented in this study shows very few sole melanophores, all being scattered throughout the sole making it appear whitish or translucent (Fig 3F). There is clearly no species-specific melanophore pattern in the juveniles. Pigmentation of the sole in *M*. *griseus* is therefore an adult feature. The Atlantic juvenile specimens examined by Cohen [19] showed similarly no sole patterns and therefore also supporting this ontogenetic development. Alternatively, the two juvenile specimens examined by Cohen could belong to the new species *M*. *griseus*, although no adult *Monacoa* specimens with the “*griseus*” sole pigmentation pattern has ever been recorded from the Atlantic Ocean, making ontogenetic transformation the most parsimonious explanation. All juveniles of the three *Monacoa* species are therefore likely to show similar whitish, strong bioluminescent soles with very few scattered melanophores present in no distinct patterns. The developing rectal bulb, a diverticulum of the gut immediately below the anus, is much more visible in the juvenile specimen of *M*. *griseus* than in the adults (see insert in Fig 3F). This feature is likely present for most opisthoproctid larvae/juveniles [48], as similar distinct heavy pigmented tissues have also been observed in *O*. *soleatus* [49], *M*. *grimaldii* [12, 47] and even more so in a 12.0 mm SL specimen tentatively referred to *Winteria telescopa* [50]. The juvenile specimen of *M*. *griseus* from this study (CBM-ZF 14757) is 17.8 mm SL and extends the development of the rectal bulb into this life stage. This is somewhat in agreement with the related argentinoids that show prolonged transformations from larvae to demersal juveniles [3]. The continuous development of the rectal light organ into the juvenile life stage is also in agreement with the other juvenile traits: uniform sole pattern, a proportionately large snout and the black latero-dorsal irregular blotch. These characters are present between 17.8 and 25.7 mm SL (Tables 2 and 3). Dissection of the post-larvae of *Rhynchohyalus natalensis* revealed an anal bulb present although absent in the adult [12, 14]. All species of *Monacoa* clearly utilize bioluminescence in both juvenile and adult life stages. However, the rectal bulb in the juvenile specimen examined during this study was much more visible than in any of the adults examined.

The orientation of the anal fin base changes through development, being almost horizontally positioned in the juveniles and transforming into the oblique or almost vertical position observed in adult *Monacoa* species. The anal fin is located on the outer posterior margin of the sole, with the transformation into adulthood resulting in a different orientation also noted by Schmidt [47] when only one juvenile specimen was known. Noteworthy, the anal fin is present in juveniles of the closely related *O*. *soleatus*, although becoming reduced, retrorse or disappears in larger specimens.

The peculiar appearance and lack of diagnostic traits for species identification in juveniles has obviously confused in the past. For example, Clarke & Wagner [51] tentatively referred a 17.0 mm specimen caught off Hawaii to *M*. *grimaldii*, although with reservations [52]. As no juveniles that unambiguously can be referred to *M*. *niger* are known and can be compared to *M*. *griseus* (CBM 14757), we note only that the Central Pacific and Hawaii waters possibly have both *M*. *niger* and *M*. *griseus* present.

### Bioluminescence in sole-bearing opisthoproctids

We here show that melanophore pigment patterns of the sole in *Monacoa* are species-specific and speculate the function a mode of communication. The mechanism appears very simple; contraction/expansion of the sole thereby polarizing outgoing light through the variable pigmented sole scales [11]. The wide sole creates a large silhouette in the water column from down-welling sunlight and a large production and regulation of light used in counter-illumination and/or communication must be taking place. The bioluminescence from the rectal light bulb is produced by the bacteria *Photobacterium phosphoreum* [53]. The rectal bulb is anatomically similar to the ceratioid (deep-sea anglerfishes) escas [11] although the types of bacteria are different [54]. Light is channeled through a lens in the bulb and into the reflector-lumen before emission. This specular system of reflection utilizing guanine crystals [55] is probably providing a strong luminescence, obliterating the intuitively problematic body contour of *Monacoa* and *Opisthoproctus*. Details of light emission regarding either communication or counter-illumination remain unknown, although likely autonomic influenced by down-welling sunlight and/or surrounding bioluminescent light production alike. Concerning species delimitation mainly from melanophore patterns at this stage, corroborated by mitogenomic data, it must be noted that Bertelsen & Munk [12] did observe some differences between their material of adult and juvenile *O*. *soleatus* and *M*. *grimaldii* pertaining to septa partitioning of the hyaloid body and associated smooth muscles. Similar work incorporating the new species described here would be interesting, as organization of the light transmitting hyaloid body is likely involved in direction and intensity of light, in conjunction with the sole melanophores acting as individual light screens. In this case, the speculated relatively simple construction of polarizing light by variation in the pigmentation of the sole scales should instead be considered a somewhat elaborate “nano-technological” innovation that controls light emission. Recently, light polarization matching (polarocrypsis) was discovered to be prevalent in open-ocean fishes, providing a superior camouflage system in a three-dimensional sunlit habitat without possibility for hiding [56]. The presence of similar cryptic advantages in mesopelagic fishes seems likely even considering depths less illuminated in the open ocean. Davis et al. [57] quantified the use of ventrally positioned photophores (counter-illumination) as compared to more lateral positioned photophores (communication) in relation to speciation, and found clear evidence of higher diversification rates in clades that have evolved species-specific patterns. They included most bioluminescent deep-sea fish groups although opisthoproctids were omitted. However, opisthoproctids show deviant bioluminescent structures as compared to other deep-sea fishes and the distinction between counter-illuminating-ventral and communication-lateral is not appropriate for these fishes. The relatively confined depth distribution of most sole-bearing opisthoproctids caught between 400–700 m indicates residual sunlight an influence. The peculiarity-factor is high in these fishes and has lead researchers to deem them “teratological” variations of other tube-eyes [58] or their looks being influenced by vitamin-deficiency [59]. It is now clear that the “depauperate” sole-bearing opisthoproctids have diversified into more species than previously known, strongly suggesting that the sole-patterns are species-specific and used in bioluminescent communication. Standard formalin fixation and ethanol preservation in museum collections is likely a factor in underestimating the true species diversity of some open-ocean deep-sea fish groups. Long-nosed mirrorbellies in the re-erected genus *Monacoa* support that communication equals speciation in the deep-sea pelagic habitats.

## Supporting Information

S1 File Jupyter Notebook illustrating the Illumina data processing and assembly of mitogenomes using MITObim. (HTML)
