# Supplementary material for: Preservation Obscures Pelagic Deep-Sea Fish Diversity: Doubling the Number of Sole-Bearing Opisthoproctids and Resurrection of the Genus Monacoa (Opisthoproctidae, Argentiniformes)
Source: PLoS One. 2016 Aug 10;11(8):e0159762. doi: 10.1371/journal.pone.0159762 (PMC4980007; doi:10.1371/journal.pone.0159762)
Supplement: S1 File — (HTML) [file pone.0159762.s001.html]

MISeq\_overlapping\_PE


## OVERVIEW of the pipeline¶

The pipeline reconstructs a mitochondrial genome from (genomic) paired end Illumina data (e.g. MISeq) given a reasonable reference seed sequence, which may be a (partial) mitochondrial genome sequence of a related taxon.

## SUMMARY of the steps performed by the pipeline¶

- Defining location of data and establishing basic file structure
- Quality trim raw Illumina data using the MIRA assembler
- Extract paired end reads after quality trimming using custom Python functions
- Merge overlapping paired end reads using FLASh
- Run MITObim pipeline to reconstruct a mitochondrial genome
- Extract relevant results

## REQUIREMENTS¶

The below pipeline requires the following programs/scripts to be in your path.

- FLASh
- MIRA assembler (pipeline was tested with MIRA 4.0.2 available from here.
- MITObim

## A DEFAULT Run¶

You need **ONLY CHANGE THE FIRST SECTION** of the following cell to match it to the data at hand.
The following cell will create a basic directory structure. Subsequently it will trim raw Illumina reads using the MIRA assembler.

All subsequent cells can be executed as they are. They will expect the particular structure established here and will require no further changes under default conditions.

In [ ]:

```
%%bash

#######################
#define variables - THIS IS THE ONLY SECTION THAT NEEDS CHANGING TO FIT YOUR DATA
#######################

#sample name
sample='O_grimaldii_s19'
#location of raw forward Illumina reads
forw="/media/chrishah/STORAGE/DATA/MITObim/Jan_Poulsen/Poulsen_data/Opgr_S19_L001_R1_001.fastq.gz"
#location of raw reverse Illumina reads
reve="/media/chrishah/STORAGE/DATA/MITObim/Jan_Poulsen/Poulsen_data/Opgr_S19_L001_R2_001.fastq.gz"
#location of fasta file used as seed for MITObim assembly
seed="/media/chrishah/STORAGE/DATA/MITObim/Jan_Poulsen/Barcodes/1356_OPGR_CO1.fasta"
#minimum length for reads after trimming
min_length=100
#number of CPUs to use in analyses
threads=5


######################
#NO MORE CHANGES NEEDED FROM HERE ON OUT IF YOU'RE HAPPY WITH A DEFAULT RUN
######################

######################
#create directory structure and symbolic links to future files
######################

mkdir 1-raw
cd 1-raw
ln -s $forw pe_raw_1.fastq.gz
ln -s $reve pe_raw_2.fastq.gz
ln -s $seed seed.fasta
cd ..

mkdir 2-read-trimming
cd 2-read-trimming
ln -s pe-trimmed-1.fastq.gz $sample\_trimmed-minlength-$min_length-pe-1.fastq.gz
ln -s pe-trimmed-2.fastq.gz $sample\_trimmed-minlength-$min_length-pe-2.fastq.gz
ln -s se-trimmed.fastq.gz $sample\_trimmed-minlength-$min_length-se.fastq.gz
cd ..

mkdir 3-read-merging
cd 3-read-merging
ln -s out.extendedFrags.fastq.gz $sample\_trimmed-minlength-$min_length-merged.fastq.gz
cd ..

mkdir 4-MITObim/
ln -s 4-MITObim/mt-candidate.fasta $sample\_mt_candidate.fasta


#############################
#Trimming Illumina reads using the MIRA assembler
############################
cd 2-read-trimming/

#write manifest file as instructions for MIRA assembler
echo "
project = $sample
job = genome,denovo,accurate
parameters = -GE:not=$threads -AS:nop=0 -NW=mrnl=0 SOLEXA_SETTINGS -CO:msr=no

readgroup = $sample\_raw
data = $forw $reve
technology = solexa
strain = $sample "> manifest.conf

#running MIRA just for trimming
mira manifest.conf &> trim.log

#extract trimmed reads
echo -e "\n\n################\n\nextracting trimmed reads\n\n" &>> trim.log
miraconvert -f maf -t fastq -C -X $min_length $sample\_assembly/$sample\_d_chkpt/readpool.maf mixed-trimmed &>> trim.log

cd ..
```

**Define** a few Python functions.

In [ ]:

```
def open_fastq(filename, r=False, w=False):
    """
    Function that opens a fastq file handle
    """
    
    import gzip
    
    if not r and not w:
        raise IOError('Either reading (r) or writing (w) needs to be specified\n')
        
    if r and w:
        raise IOError('Choose either reading (r) or writing (w) \n')
    
    if r:
        mode = 'r'
    elif w:
        mode = 'w'
    
    if filename.endswith('.gz'):
        FH = gzip.open(filename, mode+'b')
    else:
        FH = open(filename, mode)
        
    return FH

def extract_good_pairs_and_singletons(to_process, out_dir):
    """
    The function parses a fastq file (gzipped supported)
    and separates paired end from singleton reads
    """
    import gzip
    from Bio import SeqIO
    from collections import defaultdict
    
    id_dict = defaultdict(list)
    
    to_process_FH = open_fastq(to_process, r=True)
    pe_1_FH = open_fastq(out_dir+'/pe-trimmed-1.fastq.gz', w=True)
    pe_2_FH = open_fastq(out_dir+'/pe-trimmed-2.fastq.gz', w=True)
    se_FH = open_fastq(out_dir+'/se-trimmed.fastq.gz', w=True)
    
    for read in SeqIO.parse(to_process_FH, 'fastq'):
        ID = read.id[:-2]
        id_dict[ID].append(read)
        if len(id_dict[ID]) == 2:
            id_dict[ID] = sorted(id_dict[ID])
            SeqIO.write(id_dict[ID][0], pe_1_FH, 'fastq')
            SeqIO.write(id_dict[ID][1], pe_2_FH, 'fastq')
            del id_dict[ID]
            
    for ID in id_dict.keys():
        SeqIO.write(id_dict[ID][0], se_FH, 'fastq')
               
    to_process_FH.close()
    pe_1_FH.close()
    pe_2_FH.close()
    se_FH.close()
    del id_dict
```

**Extract** good read pairs for further processing after read quality trimming using the Python functions defined above.

In [ ]:

```
extract_good_pairs_and_singletons(to_process="./2-read-trimming/mixed-trimmed.fastq", out_dir='./2-read-trimming/')
```

**Merge** overlapping paired end reads using the program FLASh.

In [ ]:

```
%%bash

cd 3-read-merging
flash ../2-read-trimming/pe-trimmed-1.fastq.gz ../2-read-trimming/pe-trimmed-2.fastq.gz -z -d ./ &> flash.log
cd ..
```

**Run MITObim** pipeline to reconstruct the mt genome.

In [ ]:

```
%%bash

cd 4-MITObim
/home/chrishah/Dropbox/Github/MITObim/MITObim_1.8.pl --quick ../1-raw/seed.fasta -readpool ../3-read-merging/out.extendedFrags.fastq.gz -sample sample -ref seed -end 50 --clean &> MITObim.log
cd ..
```

**Create** symbolic links to MITObim results (assuming that MITObim has converged to a stable mt readpool we use the result from the next to last iteration).

In [ ]:

```
%%bash

cd 4-MITObim/
iteration=$(ls -1 ./ | grep "iteration" | sed 's/iteration//g' | sort -n | tail -n 2 |head -n 1)
ln -s iteration$iteration/temp_baitfile.fasta mt-candidate.fasta
ln -s iteration$iteration/sample-readpool-it$iteration.fastq mt-candidate-readpool.fastq
cd ..
```
